# Nanoscale Soft Wetting Observed in Co/Sapphire during Pulsed Laser Irradiation

**DOI:** 10.3390/nano11020268

**Published:** 2021-01-20

**Authors:** Jung Won Choi, Daseul Ham, Seonghyun Han, Do Young Noh, Hyon Chol Kang

**Affiliations:** 1 School of Materials Science and Engineering and Department of Physics and Photon Science, Gwangju Institute of Science and Technology, Gwangju 61005, Korea; coolstranger@naver.com (J.W.C.); seonghyunhan@gist.ac.kr (S.H.); 2 Department of Materials Science and Engineering, Chosun University, Gwangju 61452, Korea; gkaektmf12@naver.com

**Keywords:** soft wetting, deformation of substrate surface, pulsed laser-induced dewetting, wetting ridge, Co thin films

## Abstract

Liquid drops on deformable soft substrates exhibit quite complicated wetting behavior as compared to those on rigid solid substrates. We report on a soft wetting behavior of Co nanoparticles (NPs) on a sapphire substrate during pulsed laser-induced dewetting (PLID). Co NPs produced by PLID wetted the sapphire substrate with a contact angle near 70°, which is in contrast to typical dewetting behavior of metal thin films exhibiting contact angles greater than 90°. In addition, a nanoscale γ-Al_2_O_3_ wetting ridge about 15 nm in size and a thin amorphous Al_2_O_3_ interlayer were observed around and beneath the Co NP, respectively. The observed soft wetting behavior strongly indicates that the sapphire substrate became soft and deformable during PLID. Moreover, the soft wetting was augmented under PLID in air due to the formation of a CoO shell, resulting in a smaller contact angle near 30°.

## 1. Introduction

The nature of a substrate affects the wettability of a liquid drop on top greatly. On a rigid substrate, the contact angle of a droplet, a measure of wettability, can be predicted from Young’s equation relating the balance among the interfacial tensions involved in solid–vapor, solid–liquid, and liquid–vapor in the horizontal plane [1,2]. On the other hand, on a liquid substrate force balance, both in- and out-of-plane direction should be considered, and the Neumann’s triangle has been employed in determining the contact angle [3,4,5,6,7]. Interesting physical phenomena have been reported in the wetting on a soft deformable substrate with intermediate properties between solid and liquid [5,6,7,8,9,10,11,12,13,14,15,16,17,18,19]. For example, a wetting ridge, a microscopic protrusion of the substrate at the contact line, was reported. The deformation and the elastic restoration of the substrate fundamentally alter substrate–droplet interactions [5,6,7,8,9,10,11,12].

Dewetting of metal thin films on a solid substrate is a complicated process involving various energetic factors such as surface energy, gravity, fluid dynamics, and molecular interaction as well as kinetic limitations [20,21,22,23,24]. Theories based on atomic and molecular dynamics have predicted that a metal film thinner than a critical thickness dewetts into nanoparticles (NPs) [21,22,25,26], and models such as grooving, spinodal, and Ostwald ripening have been proposed to explain experimental observations [22,26,27,28,29]. Most of these considerations are under the assumption that the substrate remains rigid. The dewetting of metal thin films is also sensitive to ambient gas that alters the wettability and the chemical state of the resultant metal NPs on the substrate. Under an oxygen ambient, complete or partial oxidation of a metal NP might occur during dewetting, resulting in an oxide shell surrounding the NP [30,31]. Noble metals such as Au and Pt are not easily oxidized, but typical transition metals, including Co, are significantly influenced by oxidation during dewetting. Furthermore, the interfacial reactivity between the metal thin film and the substrate and the existence of an interlayer are important in determining the morphology and the wettability of the NPs because they change the interfacial energy balance greatly [32,33]. Understanding the oxidation and the presence of interfacial layers is essential to elucidate the dewetting scenario and the wettability associated with the balance of surface and interface energies.

Recently, pulsed laser irradiation has been utilized to produce metal NPs through dewetting of a thin film [22,28,34,35,36,37,38]. Pulsed lasers with pulse widths ranging from ns to fs can be used for laser induced self-assembly. An fs pulsed laser can reduce unexpected damage around the irradiated target due to reduced thermal diffusion [34], while an ns pulsed laser can effectively induce photothermal effects [22]. Thin films are momentarily heated above the melting temperature under a single ns laser pulse and melted into a liquid state, and then dewetted into nanostructures [22,34,39,40]. During the rapid cooling following the pulsed laser irradiation, the nanostructures solidify into various crystal structures where their phase diagram and kinetic limitations play key roles [34,35,39,41]. On the other hand, the morphology determined by the wetting kinematics, including liquid state surface tension and fluid dynamical effects, is typically preserved after rapid cooling. Often, truncated spherical shaped NPs are observed after a number of pulsed laser irradiations [28,34,35,42]. In many cases, therefore, the final morphology of a metal NP after pulsed laser-induced dewetting (PLID) is deeply related to the behavior of a liquid metal nano-droplet on a substrate. Thus, PLID offers a way to investigate wetting of nanoscale liquid metal droplets on a substrate.

In this study, we report on the deformation of a rigid substrate during PLID and its consequences on the wetting behavior of a metal NP. We investigated the morphology and the crystal structure of the NPs produced by the PLID of Co/sapphire (0001) thin films under vacuum and atmospheric conditions. A nanoscale γ-Al_2_O_3_ wetting ridge and an amorphous Al_2_O_3_ interlayer were observed in high-resolution transmission electron microscopy (TEM) images, which indicates that the sapphire substrate became soft and deformable during pulsed laser irradiation. Furthermore, Co NP wetted the sapphire substrate with a contact angle of 71.3°. We attribute these observations to the laser-driven fluid-like behavior of the sapphire substrate, which suppresses the dewetting of Co thin films on top. This soft wetting, wetting on a deformable substrate, was augmented under the PLID in air due to the formation of a Co-oxide shell.

## 2. Materials and Methods

The Co thin films were deposited using the electron-beam evaporation technique on a sapphire (0001) single crystal wafer on which Co thin films typically grow epitaxially. The thicknesses of the Co films were estimated to be 10 nm from X-ray reflectivity measurements. A frequency doubled Q-switched Nd:YAG laser (Quantel, Brilliant ultra100) irradiated the samples placed in a vacuum chamber. The 532 nm doubled frequency beam was separated from the beam of the fundamental frequency using a dichroic mirror and used to heat the specimens. Although the 532 nm laser was not the most suitable for light absorption of Co thin films, it is very effective to introduce the PLID of Co thin films, as previously reported [22]. The pulse duration was 8 ns, and the repetition rate was 20 Hz. The fluence of a single pulse was measured to be about 340 mJ/cm^2^. The selected fluence of 340 mJ/cm^2^ was efficient in forming wetting ridges and interfacial layers between Co thin film and sapphire substrate. It also induced dramatic morphological changes in the growth of nanostructures compared to fluences near the threshold of about 100 mJ/cm^2^ [22]. The number of pulses was controlled at 10, 20, 50, 100, and 200 pulses because the PLID of Co thin films is best represented in this series. Two sets of samples were prepared to investigate the dewetting behavior of the resulting NPs and the effect of oxidation on the microstructure. One set was irradiated under vacuum and the other under dry air. For samples irradiated in vacuum, the sample chamber was kept at 5 × 10^−5^ Torr.

Changes in the surface morphology as a function of the laser pulse were examined by scanning electron microscopy (SEM, Hitachi, S-4800, Tokyo, Japan) to determine the dewetting of Co thin films into NPs during PLID. The crystal structures of the as-deposited and the laser-irradiated samples were investigated by synchrotron X-ray diffraction (XRD) measurements that were carried out at the 5D beamline of the Pohang Light Source-II in Korea. Typical θ–2θ scan profiles were recorded. The atomic structures of individual NPs were examined by TEM (FEI, Talos F200X, Thermo Scientific, Hilsboro, OR, USA). A focused-ion beam (FIB) process was used to prepare a thin cross-section of the 200-pulsed samples irradiated under both vacuum and atmospheric conditions for TEM measurements. Prior to the FIB process, a capping layer consisting of carbon and Pt layers was deposited to protect the NPs. The elemental distribution and the chemical composition of the NPs were investigated by energy-dispersive X-ray (EDX, Super-X EDS system, Thermo Scientific, Hilsboro, OR, USA) analysis during the TEM measurements.

## 3. Results and Discussion

**Figure 1:**
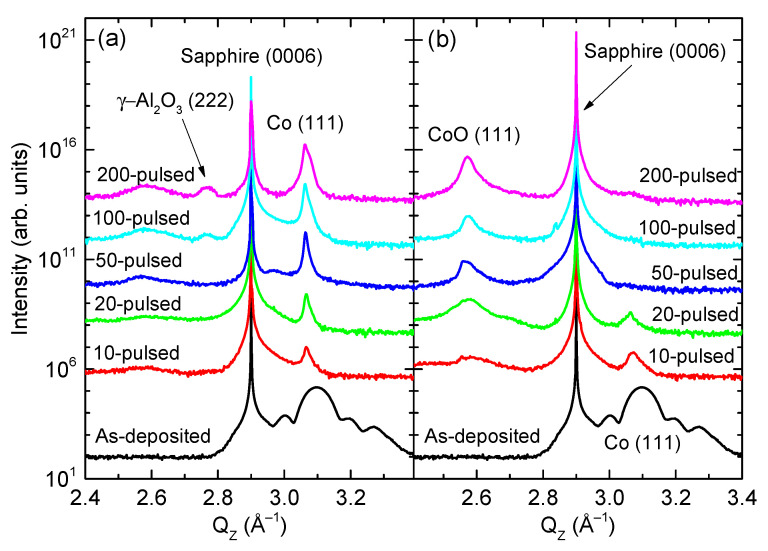
Series of XRD profiles of the samples irradiated in vacuum (**a**) and air (**b**). Observation of the γ-Al_2_O_3_ (222) peak is associated with the formation of the γ-Al_2_O_3_ wetting ridge.

Figure 1a,b shows the XRD profiles measured from the samples irradiated under vacuum and atmospheric conditions, respectively. In addition to the sharp peak detected at Q_Z_ = 2.902 Å^−1^ corresponding to the sapphire (0006) Bragg diffraction, the as-deposited Co thin films show a Co (111) Bragg peak at Q_Z_ = 3.088 Å^−1^ [(JCPDS #78-0431). The presence of an interference fringe originating from the finite film thickness of 10 nm indicates the high crystallinity of the Co thin films [43]. As predicted, the Co thin film was epitaxial to the sapphire (0001) substrate (data not shown). For samples irradiated under vacuum, the Co (111) Bragg peaks became sharp and intense, indicating that the domain size of crystalline Co increased with increasing the number of pulses. This behavior is typical of Co NPs produced using PLID. Note that a peak at Q_Z_ = 2.767 Å^−1^ was detected in the samples irradiated under vacuum that could be assigned to the γ-Al_2_O_3_ (222) Bragg diffraction, which is discussed with the TEM results later. Meanwhile, for samples irradiated under atmospheric conditions, the Co (111) Bragg peaks disappeared while the CoO (111) Bragg peaks appeared. This indicates that, during PLID, the host Co thin film became oxidized in air.

**Figure 2:**
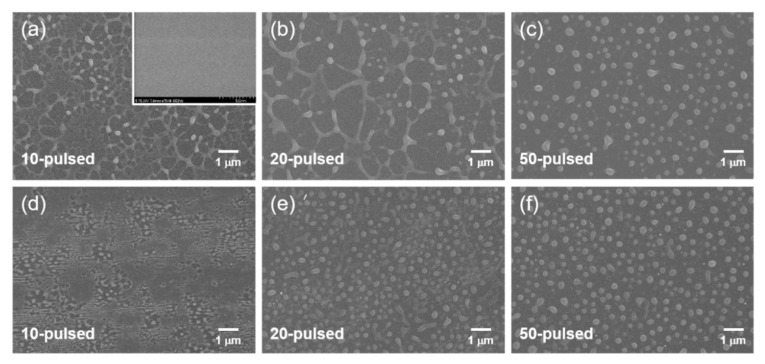
SEM images depicting the morphological evolution from a Co thin film to nanoparticles (NPs) through pulsed laser-induced dewetting (PLID), which follows the grooving model. Samples were prepared in vacuum (**a**–**c**) and air (**d**–**f**). Inset in (**a**) is SEM image of an as-deposited Co thin film.

The changes in the surface morphology as a function of the laser pulse were investigated by analyzing top-view SEM images shown in Figure 2. The samples irradiated under vacuum (Figure 2a–c) and atmospheric conditions (Figure 2d–f) show the similar surface morphological evolution exhibiting dewetting from a thin film. The dewetting process complies with the grooving model, as evidenced by the hole formation and the Rayleigh instability of the rims [22,26,35]. By analyzing the size distribution of the NPs, it was evident that the average NP diameter decreased with increasing the number of pulses under vacuum but increased under air. The former can be explained by the fragmentation and the evaporation of the NPs [22,44], while the latter case is associated with the effects of oxidation, including the Kirkendall effect [45,46].

**Figure 3:**
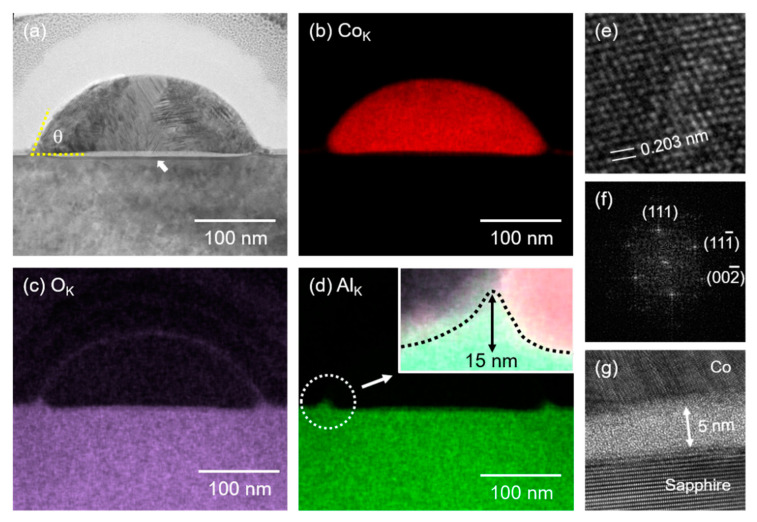
TEM results of an individual Co NP in the 200-pulsed sample irradiated under vacuum. (**a**) Low-magnification TEM image. Corresponding energy-dispersive X-ray (EDX) mapping images of Co_K_ (**b**), O_K_ (**c**), and Al_K_ (**d**) emissions are illustrated. The inset in (**d**) shows the left corner of the wetting ridge near the contact line obtained with a higher magnification. The wetting ridge consists of Al and O. (**e**) High-resolution TEM image. (**f**) Fast Fourier transform (FFT) pattern of the Co crystalline domain corresponding to (**e**). (**g**) High-resolution TEM image highlights the amorphous Al_2_O_3_ interlayer between the Co NP and sapphire substrate.

The morphology of the Co NPs exhibited signatures of the soft wetting, the wetting phenomena observed on a deformable substrate. Figure 3 shows a TEM image together with the EDX mappings of Co_K_, O_K_, and Al_K_ emissions of a 200-pulsed sample. The NP obtained by the PLID in vacuum was a pure Co NP in the overall shape of truncated-hemisphere, as illustrated by the Co_K_ mapping image in Figure 3b and by the lattice image and the corresponding fast Fourier transform (FFT) pattern shown in Figure 3e,f, respectively, as expected in a typical PLID of a metal film. The Co NP shows a polycrystalline microstructure consisting mostly of the <111> domains oriented along the surface normal direction with an atomic layer spacing of approximately 0.203 nm. Remarkably, however, we found a few features of the soft wetting at the boundary separating the Co NP and the sapphire substrate. First of all, the substrate was deformed into a shape similar to the one predicted in the macroscopic theories of soft wetting [5,6,7,8,9,10,11,12,33], as indicated by the arrow in Figure 3a. The length scale of deformation, however, was only about 5 nm. Furthermore, we found a wetting ridge, the concave meniscus located at the contact line where the Co NP, the substrate, and the vacuum space meet, as highlighted in the inset of Figure 3d (also in Figures S1 and S2 in the Supplementary Material). The wetting ridge was mostly aluminum oxide, as confirmed in the EDX images shown in Figure 3b–d. The height of the wetting ridge was approximately 15 nm.

Generally, the deformation of a substrate has been reported in liquid droplets on soft substrates including liquids and gels [5,6,7,8,9,10,11,12,13,14,15,16,17,18,19]. The existence of the wetting ridge and the substrate deformation indicates that the sapphire substrate, which was originally rigid, became soft during the pulsed laser irradiation and exhibited fluid-like behavior. According to a thermal modeling of Co thin films on SiO_2_ supported by experimental data [22], the melting threshold laser fluence of a 10-nm-thick Co film is about 100 mJ/cm^2^, which is less than the fluence employed in this study. The Co NPs formed after a number of pulsed laser irradiations were presumed to remain in a liquid state for a few tens of ns after following pulse irradiations [22,34,39,40,41]. Furthermore, the sapphire near the interfacial region to Co NPs was likely to melt momentarily or to become soft. An 11-nm-thick Co/SiO_2_ is predicted to reach about 2500 K under a fluence of 100 mJ/cm^2^, which is even above the melting point of sapphire [22]. The 5-nm-thick interfacial layer distinguishable in Figure 3a suggested that the top surface of the sapphire be liquified momentarily, which was frozen into an amorphous interfacial layer during rapid cooling, as supported by a high resolution TEM image shown in Figure 3g. It is noted that the morphological changes such as wetting ridge and interfacial layer formation of sapphire substrate were mostly affected by thermal energy driven by photothermal heating of Co NPs. The direct absorption of the 532 nm laser on the sapphire substrate was negligible.

**Figure 4:**
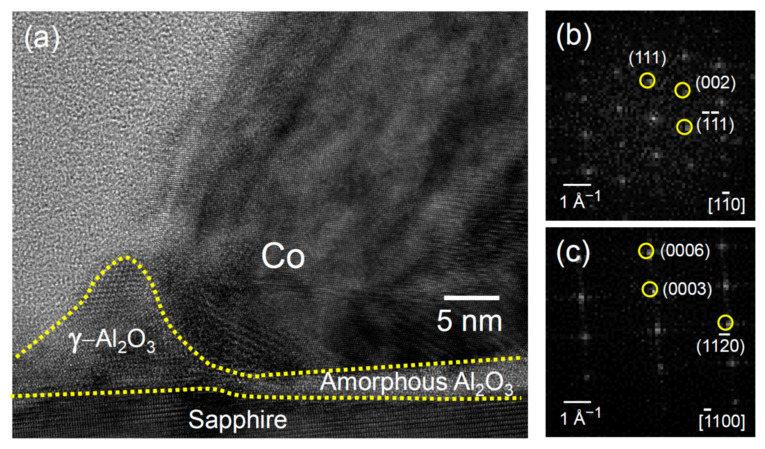
(**a**) TEM image illustrating the region near the contact line that exhibits the deformation of substrate signified by the γ-Al_2_O_3_ wetting ridge and the amorphous Al_2_O_3_ interlayer. (**b**) FFT pattern of the γ-Al_2_O_3_ crystal confirming its face centered cubic (FCC) structure with a lattice constant of 0.79 nm. (**c**) FFT pattern of the crystalline sapphire substrate for comparison. Scale bar represents the reciprocal space unit.

We interpret that the observed wetting ridge magnified in Figure 4a was due to the deformation of the softened sapphire substrate induced by the Co liquid nano-droplet whose shape was mostly preserved during rapid cooling after the pulsed laser irradiation. We think that the liquid aluminum oxide was pulled up by the capillary force at the contact line between the Co nano-droplet and the sapphire substrate, which formed the wetting ridge. In addition, the formation of the wetting ridge can be discussed in terms of atomic diffusion in the liquid state or grooving through defects at the Co/sapphire interface during PLID. These are associated with very small elastic distortion and local diffusion to equilibrate the energy balance at the triple line [47,48,49]. When the Co NPs on the sapphire surface were heated to melting point of Co NPs, the impurities could also cooperate with the Al vacancies on sapphire substrates. This improved the diffusivity of alumina to form wetting ridges during pulsed laser irradiation [47,48,49]. Moreover, the amorphous Al_2_O_3_ interlayer showed double-convex shape due to higher surface energy compared to Co and sapphire substrate [50].

The deformation of the substrate suppressed the dewetting of Co NP, which was signified by the contact angle smaller than 90°, as indicated in Figure 3a. The contact angle (θ), estimated using a simple relation, *h* = *r* (1 − cosθ), where *h* (102 nm) and *r* (150 nm) are the height and the radius of the hemisphere, respectively, was about 71.3°, indicating that the system was in the wetting (θ < 90°) rather than the dewetting (θ > 90°) regime. This value is significantly smaller than the contact angle of noble metal NPs such as Au and Pt produced in PLID, which are usually larger than 110° [34,42,51].

The wetting ridge froze into the crystalline γ-Al_2_O_3_ phase during rapid cooling following a laser irradiation, which is in contrast to the fact that the interlayer became amorphous. As shown in Figure 4a, atomic planes of 0.455 nm spacing were observed, corresponding to the (111) planes of γ-Al_2_O_3_ crystal. The FFT pattern shown in Figure 4b exhibits the diffraction pattern of a face centered cubic (FCC) structure with a lattice constant of 0.79 nm in the [1$\bar{1}$0] zone axis, which also confirms the existence of the γ-Al_2_O_3_ phase. For comparison, the FFT pattern of the sapphire substrate displayed in Figure 4c shows the symmetry of α-Al_2_O_3_ clearly distinguished from that of the γ-Al_2_O_3_. The presence of the γ-Al_2_O_3_ crystals is consistent with the XRD results (Figure 1a).

**Figure 5:**
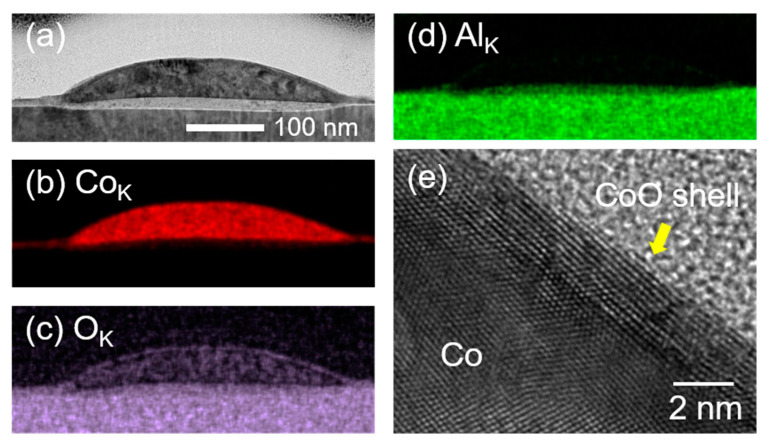
(**a**) TEM image of an individual NP in a 200-pulsed sample irradiated in air. The amorphous Al_2_O_3_ interlayer is clearly observed. Corresponding EDX mapping images of Co_K_ (**b**), O_K_ (**c**), and Al_K_ (**d**) emissions are illustrated. (**e**) High-resolution TEM image illustrating a CoO shell and Co crystal domains at the boundary of the NP.

The soft wetting behavior was more pronounced in the NPs produced by a PLID of a Co film in air, as illustrated in the TEM and the EDX mapping images in Figure 5. A thin truncated hemispherical NP is observed in the TEM image in Figure 5a, which also shows the deformation of the sapphire surface associated with an amorphous Al_2_O_3_ interlayer similar to the Co NPs produced in vacuum. The EDX mapping analysis shown in Figure 5b–d, especially the O_K_ emission (Figure 5c), shows not only the formation of the CoO shell but also the localized CoO domains within the NP. The high-resolution TEM image shown in Figure 5e represents the atomic structure of the CoO shell and the Co domains in the NP. The thickness of the CoO shell was estimated to be approximately 2 nm. The formation of a CoO shell indicates an oxidation process via the Kirkendall effect [45,46]. The CoO shell passivates the surface, preventing the diffusion of oxygen atoms into the core at an early stage. On the other hand, Co atoms in the core diffuse into the surface of the CoO shell and are then oxidized, which results in an increase in the diameter of the NPs, consistent with the SEM results in Figure 2. In contrast to the CoO NPs formed via thermal annealing through the Kirkendall effect [45,46], no voids or CoO domains were present in the core NPs. It is worth noting that void formation during the nanoscale Kirkendall effect is a nucleation-type phenomenon that occurs only when the oxide shell is thicker than its critical thickness [52]. We also assumed that the 200-pulsed sample showed the initial oxidation state of Co NPs rather than a fully oxidized state. As a result, the CoO shell observed in this paper was too thin to initiate hollowing.

To estimate the wettability of NPs produced under atmospheric conditions, θ = 30.7° was calculated using the values *h* = 56 nm and *r* = 400 nm. This value is significantly smaller than the contact angle of 71.3° for Co NPs (Figure 3). The height of the wetting ridge was also reduced to about 10 nm. This can be attributed to the combined role of the CoO shell and the deformation of the substrate surfaces. We believe that the oxidation of NPs significantly suppressed the dewetting of Co thin films during PLID. In other words, the initially formed CoO shell (a thin capping layer of 2 nm in this study) served as a frame for dewetting, which ultimately led to a smaller contact angle. This is conceptually similar to previous reports on the effective prevention of dewetting using a capping layer [31,53,54].

## 4. Conclusions

In summary, we observed soft wetting behaviors in the Co NPs on a sapphire substrate produced by PLID. NPs produced by PLID both in vacuum and in air wetted the sapphire substrate with contact angles of 71.3° and 30.7°, respectively. We found that high-power pulsed laser irradiation softened the sapphire substrate, and the presence of a Co nano-droplet introduced the deformation of sapphire substrates signified by a nanoscale γ-Al_2_O_3_ wetting ridge as well as a thin amorphous Al_2_O_3_ interlayer. The deformation of the substrate surfaces was critical in suppressing the dewetting of the resultant NPs in PLID. Furthermore, the CoO shell formed by oxidation played an important role in making the contact angle of CoO NPs smaller. We expect the soft wetting behavior reported here to be observed in other metal droplets during PLID. Co NPs with controlled size distribution can be implemented for surface-enhanced plasmonic applications and high-sensitivity magnetic devices. Co oxide NPs can also be applied to batteries, as they have a lot of oxygen vacancies that can improve redox chemical activity. This study provides a guide for studying various phenomena in laser–matter interactions, such as nano-bubbling and complex fluid-like behaviors in nanostructures.

## Data Availability

The data presented in this study are available on request from the corresponding author.

## Supplementary Materials

The following are available online at https://www.mdpi.com/2079-4991/11/2/268/s1, Figure S1: (a) Low-magnification scanning TEM image and (b) EDX mapping image of a Co NP. Wetting ridges are clearly observed at the contact line. Figure S2: TEM and EDX mapping images highlighting the left corner of the wetting ridge near the contact line. (a) Scanning TEM image. EDX mapping images of (b) Co_K_, (c) O_K_, (d) Al_K_ emissions. The color map in (e) clearly shows the wetting ridge.

## References

[1] Young T. (1805). An essay on the cohesion of fluids. Phil. Trans. R. Soc..

[2] Makkonen L. (2016). Young’s equation revisited. J. Phys. Condens. Matter.

[3] Neumann F. (1894). Vorlesungen Uber Die Theorie der Capillaritt.

[4] De Gennes P.-G., Brochard-Wyart F., Quere D. (2010). Capillarity and Wetting Phenomena: Drops, Bubbles, Pearls, Waves.

[5] Marchand A., Das S., Snoeijer J.H., Andreotti B. (2012). Contact angles on a soft solid: From Young’s law to Neumann’s law. Phys. Rev. Lett..

[6] Style R.W., Dufresne E.R. (2012). Static wetting on deformable substrates, from liquids to soft solids. Soft Matter.

[7] Jerison E.R., Xu Y., Wilen L.A., Dufresne E.R. (2011). Deformation of an elastic substrate by a three-phase contact line. Phys. Rev. Lett..

[8] Sadullah M.S., Semprebon C., Kusumaatmaja H. (2018). Drop dynamics on liquid-infused surfaces: The role of the lubricant ridge. Langmuir.

[9] Leong F.Y., Le D.-V. (2020). Droplet dynamics on viscoelastic soft substrate: Toward coalescence control. Phys. Fluids.

[10] Van Gorcum M., Karpitschka S., Andreotti B., Snoeijer J.H. (2020). Spreading on viscoelastic solids: Are contact angles selected by Neumann’s law?. Soft Matter.

[11] Karpitschka S., Das S., Van Gorcum M., Perrin H., Andreotti B., Snoeijer J.H. (2015). Droplets move over viscoelastic substrates by surfing a ridge. Nat. Commun..

[12] De Pascalis R., Dervaux J., Ionescu I., Limat L. (2018). Numerical multiscale modelling of nonlinear elastowetting. Eur. J. Mech. A.

[13] Roy R., Seiler R.L., Weibel J.A., Garimella S.V. (2020). Soft surface: Droplets on soft surfaces exhibit a reluctance to coalesce due to an intervening wetting ridge. Adv. Mater. Interfaces.

[14] Dey R., Daga A., DasGupta S., Chakraborty S. (2015). Electrically modulated dynamic spreading of drops on soft surfaces. Appl. Phys. Lett..

[15] Park S.J., Weon B.M., Lee J.S., Lee J., Kim J., Je J.H. (2014). Visualization of asymmetric wetting ridges on soft solids with X-ray microscopy. Nat. Commun..

[16] Schellenberger F., Xie J., Encinas N., Hardy A., Klapper M., Papadopoulos P., Butt H.-J., Vollmer D. (2015). Direct observation of drops on slippery lubricant-infused surfaces. Soft Matter.

[17] Pu G., Severtson S.J. (2011). Dependence of wetting behavior on the thickness of highly viscoelastic films. J. Phys. Chem. C.

[18] Karpitschka S., Pandey A., Lubbers L.A., Weijs J.H., Botto L., Das S., Andreotti B., Snoeijer J.H. (2016). Liquid drops attract or repel by the inverted Cheerios effect. Proc. Natl. Acad. Sci. USA.

[19] Carre A., Gastel J.-C., Shanahan M.E.R. (1996). Viscoelastic effects in the spreading of liquids. Nature.

[20] Bischof J., Scherer D., Herminghaus S., Leiderer P. (1996). Dewetting modes of thin metallic films: Nucleation of holes and spinodal dewetting. Phys. Rev. Lett..

[21] Sharma A., Ruckenstein E. (1990). Energetic criteria for the breakup of liquid films on nonwetting solid surfaces. J. Colloid Interface Sci..

[22] Trice J., Thomas D., Favazza C., Sureshkumar R., Kalyanaraman R. (2007). Pulsed-laser-induced dewetting in nanoscopic metal films: Theory and experiments. Phys. Rev. B.

[23] Peschka D., Haefner S., Marquant L., Jacobs K., Münch A., Wagner B. (2019). Signatures of slip in dewetting polymer films. Proc. Natl. Acad. Sci. USA.

[24] Lee J., Pandey P., Sui M., Li M.-Y., Zhang Q., Kunwar S. (2015). Evolution of self-assembled Au NPs by controlling annealing temperature and dwelling time on sapphire (0001). Nanoscale Res. Lett..

[25] Pahlavan A.A., Cueto-Felgueroso L., Hosoi A.E., McKinley G.H., Juanes R. (2018). Thin films in partial wetting: Stability, dewetting and coarsening. J. Fluid Mech..

[26] Gentili D., Foschi G., Valle F., Cavallini M., Biscarini F. (2012). Applications of dewetting in micro and nanotechnology. Chem. Soc. Rev..

[27] Krishna H., Sachan R., Strader J., Favazza C., Khenner M., Kalyanaraman R. (2010). Thickness-dependent spontaneous dewetting morphology of ultrathin Ag films. Nanotechnology.

[28] Fowlkes J.D., Kondic L., Diez J., Wu Y., Rack P.D. (2011). Self-assembly versus directed assembly of nanoparticles via pulsed laser induced dewetting of patterned metal films. Nano Lett..

[29] Naffouti M., Backofen R., Salvalaglio M., Bottein T., Lodari M., Voigt A., David T., Benkouider A., Fraj I., Favre L. (2017). Complex dewetting scenarios of ultrathin silicon films for large-scale nanoarchitectures. Sci. Adv..

[30] Gazit N., Richter G., Sharma A., Klinger L., Rabkin E. (2019). Engineering of hollow AlAu2 nanoparticles on sapphire by solid state dewetting and oxidation of Al. Mater. Des..

[31] Herz A., Franz A., Theska F., Hentschel M., Kups T., Wang D., Schaff P. (2016). Solid-state dewetting of single- and bilayer Au-W thin films: Unraveling the role of individual layer thickness, stacking sequence and oxidation on morphology evolution. AIP Adv..

[32] Monestrol H., Schmirgeld-Mignot L., Poissonnet S., Lebourgeois C., Martin G. (2003). Reactive solid state dewetting: Interfacial cavitation in the system Ag-Ni-O. Interface Sci..

[33] Hsieh J.Y., Chen J.L., Chen C., Lin H.C., Yang S.S., Hwang C.C. (2010). Reactive wetting behaviors of Sn/Cu systems: A molecular dynamics study. Nanomicro Lett..

[34] Makarov S.V., Milichko V.A., Mukhin I.S., Shishkin I.I., Zuev D.A., Mozharov A.M., Krasnok A.E., Belov P.A. (2016). Controllable femtosecond laser-induced dewetting for plasmonic applications. Laser Photonics Rev..

[35] Kondic L., González A.G., Diez J.A., Fowlkes J.D., Rack P. (2020). Liquid-state dewetting of pulsed-laser-heated nanoscale metal films and other geometries. Annu. Rev. Fluid Mech..

[36] Letfullin R.R., Joenathan C., George T.F., Zharov V.P. (2006). Laser-induced explosion of gold nanoparticles: Potential role for nanophotothermolysis of cancer. Nanomedicine.

[37] Singer J.P., Lin P.-T., Kooi S.E., Kimerling L.C., Michel J., Thomas E.L. (2013). Direct-write thermocapillary dewetting of polymer thin films by a laser-induced thermal gradient. Adv. Mater..

[38] Bornemann S., Yulianto N., Spende H., Herbani Y., Prades J.D., Wasisto H.S., Waag A. (2020). Femtosecond Laser Lift-Off with Sub-Bandgap Excitation for Production of Free-Standing GaN Light-Emitting Diode Chips. Adv. Eng. Mater..

[39] Rack P.D., Guan Y., Fowlkes J.D., Melechko A.V., Simpson M.L. (2008). Pulsed laser dewetting of patterned thin metal films: A means of directed assembly. Appl. Phys. Lett..

[40] Yadavali S., Khenner M., Kalyanaraman R. (2013). Pulsed laser dewetting of Au films: Experiments and modeling of nanoscale behavior. J. Mater. Res..

[41] Duff W.H., Zhigilei L.V. (2007). Computational study of cooling rates and recrystallization kinetics in short pulse laser quenching of metal targets. J. Phys. Conf. Ser..

[42] Ruffino F., Pugliara A., Carria E., Bongiorno C., Spinella C., Grimaldi M.G. (2012). Formation of nanoparticles from laser irradiated Au thin film on SiO_2_/Si: Elucidating the rayleigh instability role. Mater. Lett..

[43] Warren B.E. (1990). X-ray Diffraction.

[44] Werner D., Furube A., Okamoto T., Hashimoto S. (2011). Femtosecond laser-induced size reduction of aqueous gold nanoparticles: In situ and pump−probe spectroscopy investigations revealing Coulomb explosion. J. Phys. Chem. C.

[45] Ha D.-H., Moreau L.M., Honrao S., Hennig R.G., Robinson R.D. (2013). The oxidation of cobalt nanoparticles into Kirkendall-hollowed CoO and Co_3_O_4_: The diffusion mechanisms and atomic structural transformations. J. Phys. Chem. C.

[46] Zhang D., Jin C., Li Z.Y., Zhang Z., Li J. (2017). Oxidation behavior of cobalt nanoparticles studied by in situ environmental transmission electron microscopy. Sci. Bull..

[47] Chatain D., Galy D. (2006). Interfaces between Pb grains and Cu surfaces. J. Mater. Sci..

[48] Saiz E., Tomsia A.P., Cannon R.M. (1998). Ridging effects on wetting and spreading of liquids and solids. Acta Mater..

[49] Ghetta V., Chatain D. (2002). Morphologies adopted by Al_2_O_3_ single-crystal surfaces in contact with Cu droplets. J. Am. Ceram. Soc..

[50] Klinger L., Rabkin E. (2013). Sintering of spherical particles of two immiscible phases controlled by surface and interphase boundary diffusion. Acta Mater..

[51] Ahn K., Lee S.Y., Cho I.H., Kim Y., Kang H.C., Noh D.Y. (2020). Phase separated bi-metallic nnaoparticles formed by pulsed laser dewetting. Nanotechnology.

[52] Klinger L., Rabkin E. (2015). On the nucleation of pores during the nanoscale Kirkendall effect. Mater. Lett..

[53] Cao P., Bai P., Omrani A.A., Xiao Y., Meaker K.L., Tsai H.-Z., Yan A., Jung H.S., Khajeh R., Rodgers G.F. (2017). Preventing thin film dewetting via graphene capping. Adv. Mater..

[54] Su D., Yu M., Zhang G., Jiang S., Qin Y., Li M. (2020). Highly thermally stable Au–Al bimetallic conductive thin films with a broadband transmittance between UV and NIR regions. J. Mater. Chem. C.

