# Supplementary material for: Nanoscale Soft Wetting Observed in Co/Sapphire during Pulsed Laser Irradiation
Source: Nanomaterials (Basel). 2021 Jan 20;11(2):268. doi: 10.3390/nano11020268 (PMC7909543; doi:10.3390/nano11020268)
Supplement: Supplementary file 1 [file nanomaterials-11-00268-s001.pdf]

Supplementary materials:

Nanoscale soft wetting observed in Co/Sapphire during pulsed laser irradiation

Jung Won Choi[1], Daseul Ham[2], Seonghyun Han[1], Do Young Noh[1,]*, Hyon Chol Kang[2,]*

[1]School of Materials Science and Engineering, Department of Physics and Photon Science, Gwangju Institute of Science and Technology, Gwangju, 61005, Korea

[2]Department of Materials Science and Engineering, Chosun University, Gwangju 61452, Korea

*E-mail: dynoh@gist.ac.kr, kanghc@chosun.ac.kr

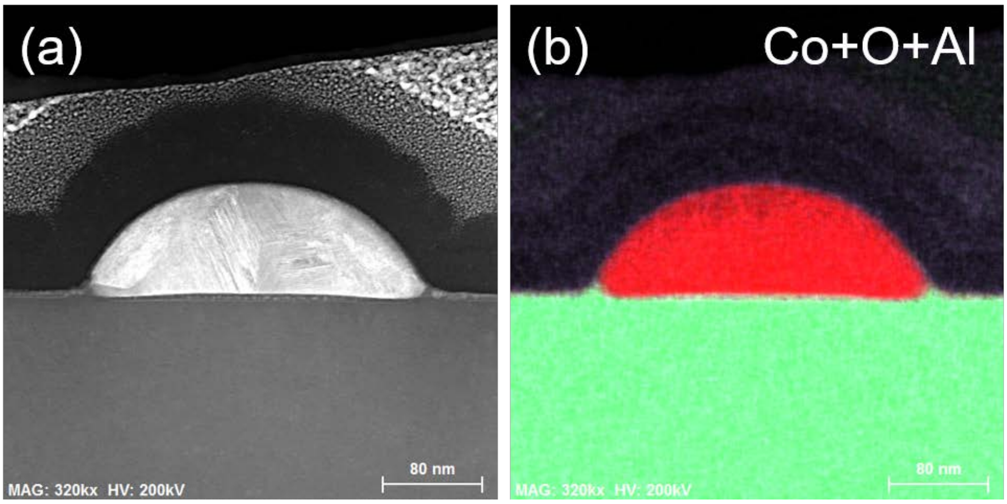

Figure S1. (a) Low-magnification scanning TEM image and (b) EDX mapping image of a Co NP. Wetting ridges are clearly observed at the contact line.

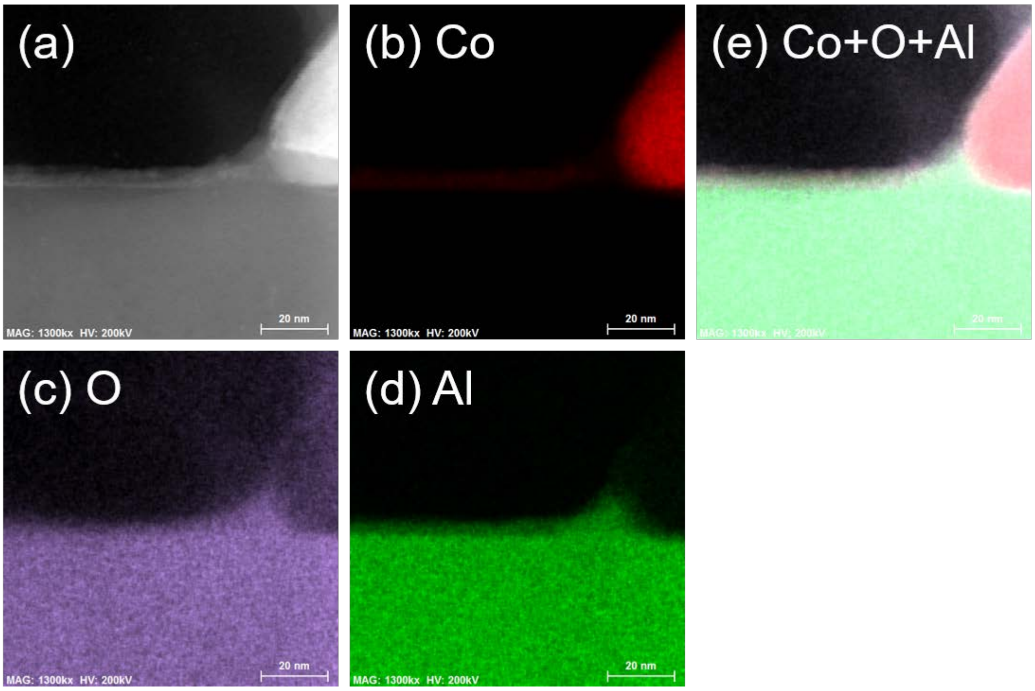

Figure S2. TEM and EDX mapping images highlighting the left corner of the wetting ridge near the contact line. (a) Scanning TEM image. EDX mapping images of (b) Co_K, (c) O_K, (d) Al_K emissions. The color map in (e) clearly shows the wetting ridge.
